# Associations of visceral adiposity index and lipid accumulation product with overactive bladder among U.S. adults: a cross-sectional study

**DOI:** 10.1097/JS9.0000000000002666

**Published:** 2025-06-12

**Authors:** Jinbao Wang, Qiyou Wu, Bo Chen, Jinze Li, Xinyang Liao, Qiang Wei

**Affiliations:** a Department of Urology, West China Hospital, Sichuan University, Chengdu, Sichuan Province, China; b West China School of Medicine, Sichuan University, Chengdu, Sichuan Province, China

The aim of this study was to explore the correlation between visceral adiposity index (VAI) or lipid accumulation product (LAP) index and the prevalence of overactive bladder (OAB). Our article is fully compliant with the TITAN 2025 Guidelines regarding the statement and use of artificial intelligence^[1]^. No AI was used in the generation of this article.

**HIGHLIGHTS**

- Explored the relationships between VAI or LAP and OAB.
- Both VAI and LAP were positively correlated with OAB.
- Age and gender had an effect on the relationship between VAI or LAP and OAB.
- A large sample study that explored the correlation between VAI or LAP and OAB.

We analyzed data from the National Health and Nutrition Examination Survey (NHANES) 2005–2016. VAI and LAP were calculated by laboratory tests and physical examinations with subsequent ln conversion, whereas OAB was diagnosed by self-reported questionnaire. Multiple logistic regression, nonlinear correlation analysis, stratified analysis, and interaction tests were used for statistical analysis.

A total of 27 144 participants were included in this study, 5410 of whom were diagnosed with OAB. OAB prevalence increased with higher VAI and LAP quartiles. After full adjustment for covariates, a one-unit increase in ln-transformed VAI (odds ratio (OR) = 1.097, 95% confidence interval (CI) = 1.049–1.147, *P* = 0.00005) or LAP (OR = 1.272, 95% CI = 1.219–1.328, *P* < 0.00001) remained significantly associated with OAB (Table 1). Smooth curve fitting showed that VAI for ln-transformed was linearly correlated with OAB, whereas LAP was curvilinear (Fig. 1). Stratified analyses demonstrated significant interactions between both indices and age, sex, and comorbidities for both VAI and LAP (Fig. 2).

**Figure 1.**
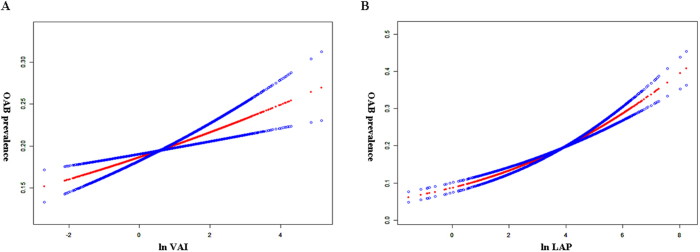
**(A)** Linear association between VAI and the prevalence of OAB. (**B)** Smooth curve fitting for LAP and OAB correlations.

**Figure 2.**
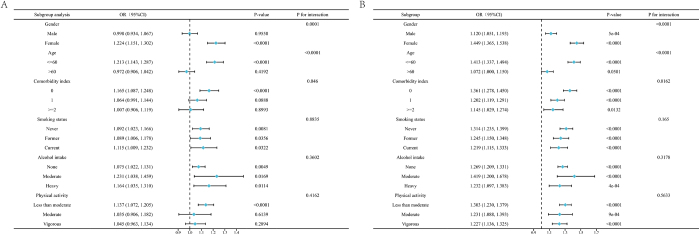
**(A)** Subgroup analysis between VAI and OAB prevalence. (**B)** Subgroup analysis between LAP and OAB prevalence.

**Table 1:** Multivariate regression model of the relationship between VAI or LAP and the prevalence of OAB

Exposure	Model 1		Model 2		Model 3	
	OR (95%CI)	*P* value	OR (95%CI)	*P* value	OR (95%CI)	*P* value
VAI						
Continuous	1.195 (1.153, 1.239)	<0.00001	1.208 (1.158, 1.260)	<0.00001	1.097 (1.049, 1.147)	0.00005
Q1	Ref		Ref		Ref	
Q2	1.209 (1.107, 1.320)	0.00002	1.159 (1.050, 1.278)	0.00327	1.047 (0.945, 1.160)	0.3796
Q3	1.396 (1.280, 1.522)	<0.00001	1.309 (1.187, 1.444)	<0.00001	1.137 (1.027, 1.260)	0.01386
Q4	1.493 (1.370, 1.626)	<0.00001	1.481 (1.342, 1.634)	<0.00001	1.192 (1.075, 1.322)	0.00088
*P* for trend	<0.00001		<0.00001		0.00028	
LAP						
Continuous	1.412 (1.364, 1.462)	<0.00001	1.409 (1.353, 1.467)	<0.00001	1.272 (1.219, 1.328)	<0.00001
Q1	Ref		Ref		Ref	
Q2	1.587 (1.444, 1.743)	<0.00001	1.385 (1.247, 1.537)	<0.00001	1.227 (1.101, 1.368)	0.00022
Q3	2.066 (1.886, 2.263)	<0.00001	1.766 (1.594, 1.955)	<0.00001	1.497 (1.346, 1.666)	<0.00001
Q4	2.329 (2.128, 2.548)	<0.00001	2.191 (1.980, 2.426)	<0.00001	1.714 (1.539, 1.908)	<0.00001
*P* for trend	<0.00001		<0.00001		<0.00001	

VAI, visceral adiposity index; LAP, lipid accumulation product index.

Model 1 adjust for: none.

Model 2 adjust for: gender, age, ethnicity, ratio of family income to poverty, education level, and marital.

Model 3 adjust for: gender, age, ethnicity, ratio of family income to poverty, education level, marital, comorbidity index, smoking status, alcohol intake, and physical activity.

In conclusion, our study shows that there was a positive correlation between VAI or LAP and the prevalence of OAB among U.S. adults, which suggests that elevated VAI or LAP indices may contribute to higher OAB risk.

## Data Availability

The data were obtained from the NHANES website (https://www.cdc.gov/nchs/nhanes/) and are completely public and free.
